# Imaging of focal seizures with Electrical Impedance Tomography and depth electrodes in real time

**DOI:** 10.1016/j.neuroimage.2021.117972

**Published:** 2021-07-01

**Authors:** Anna Witkowska-Wrobel, Kirill Aristovich, Abbe Crawford, Justin D. Perkins, David Holder

**Affiliations:** a Medical Physics and Biomedical Engineering, University College London, UK; b Royal Veterinary College, Hawkshead Lane, North Mymms, Hatfield, Hertfordshire AL9 7TA, UK

**Keywords:** EIT, Epilepsy, Seizure model, Seizure imaging, Intracranial electrodes

## Abstract

Intracranial EEG is the current gold standard technique for localizing seizures for surgery, but it can be insensitive to tangential dipole or distant sources. Electrical Impedance Tomography (EIT) offers a novel method to improve coverage and seizure onset localization. The feasibility of EIT has been previously assessed in a computer simulation, which revealed an improved accuracy of seizure detection with EIT compared to intracranial EEG. In this study, slow impedance changes, evoked by cell swelling occurring over seconds, were reconstructed in real time by frequency division multiplexing EIT using depth and subdural electrodes in a swine model of epilepsy. EIT allowed to generate repetitive images of ictal events at similar time course to fMRI but without its significant limitations. EIT was recorded with a system consisting of 32 parallel current sources and 64 voltage recorders. Seizures triggered with intracranial injection of benzylpenicillin (BPN) in five pigs caused a repetitive peak impedance increase of 3.4 ± 1.5 mV and 9.5 ± 3% (N =205 seizures); the impedance signal change was seen already after a single, first seizure. EIT enabled reconstruction of the seizure onset 9 ± 1.5 mm from the BPN cannula and 7.5 ± 1.1 mm from the closest SEEG contact (*p*<0.05, *n* =37 focal seizures in three pigs) and it could address problems with sampling error in intracranial EEG. The amplitude of the impedance change correlated with the spread of the seizure on the SEEG (*p* <<0.001, *n* =37). The results presented here suggest that combining a parallel EIT system with intracranial EEG monitoring has a potential to improve the diagnostic yield in epileptic patients and become a vital tool in improving our understanding of epilepsy.

## Highlights

• EIT was used to record ictal activity in porcine brain using depth-electrodes.
• Ictal activity caused a repetitive pattern of slow, positive impedance changes.
• EIT with depth-electrodes can image onset and spread of focal seizures in real time.
• EIT and SEEG can be recorded in parallel to optimally localize seizure onset.

## Introduction

### Background

Epilepsy affects approximately 50 million people worldwide and has a burden of premature death and residual disability (Beghi et al., 2019). Although many underlying mechanisms can lead to epilepsy, the cause of the disorder is still unknown in more than 50% of cases (Neligan et al., 2012). It is estimated that at least 30% of patients continue to have seizures despite pharmacological treatment and the likelihood of achieving seizure freedom declines with consecutive drug regimens (Neligan et al., 2012). In focal epilepsies, a resective surgery can be a highly effective treatment; however, careful localization of clearly identifiable ictal network is a prerequisite for a successful outcome (Bell et al., 2017). Currently, seizure remission during the 10-year follow-up have been reported for between 50 and 70% of the patients, who undergone the surgery (Engel, 1996; De Tisi et al., 2011). These figures are particularly low for those with extratemporal resections, for whom the reported 5-year seizure freedom varied between 14 and 33% (Bell et al., 2017; McIntosh et al., 2012,^2004^; Jobst and Cascino, 2015 2004), indicating a clear need for further improvements in presurgical assessment and surgical treatment.

One of the main limitations in presurgical assessment is an absence of a functional imaging method that would allow monitoring of the seizure onset and spread in real time. At present, identification of the seizure onset zone for surgical resection relies on a basic clinical examination, ictal semiology, neurophysiological video-telemetry EEG assessment and structural imaging with the MRI epilepsy protocol (Bell et al., 2017). The EEG assessment can be performed with scalp electrodes, however it has been shown that the precise localization of the seizure onset zone can be greatly improved with the use of intracranial EEG (iEEG, ECoG, electrocorticogram or SEEG, stereo-EEG, depth electrodes), in which the recording contacts are closer to the seizure source thus resulting in better spatiotemporal resolution compared with scalp EEG and, in some cases, allowing identification sources that would not be visible with scalp EEG at all (Schindler et al., 2016; Mullin et al., 2016). Still, neither of the methods provide imaging data and they are limited by the electrode orientation, amplitude and distance from the signal generator, and therefore potentially blind to some sources (Lachaux et al., 2003; Ebersole, 1991; von Ellenrieder et al., 2012). The sudden and dynamic nature of epileptic seizures makes some methods such as fMRI almost impossible to employ and severely limits use of others, such as ictal-SPECT or interictal-PET, which require complicated patient preparation together with constant access to expensive equipment and presence of trained personnel without a promise of any diagnostic outcome. For these reasons, there is currently no satisfactory method to image seizure onset and its propagation in real time and no single modality appears clearly superior or widely used in the clinic, especially for patients without focal lesions in MRI (Knowlton, 2006; Kaiboriboon et al., 2012; Lenkov et al., 2013). Therefore, in this study we propose to harness a new imaging method, Electrical Impedance Tomography, adopting intracranial electrodes that are already used for presurgical SEEG monitoring and, in so doing, image real time seizure onset and propagation *in vivo.* The technique is proposed to be used, at least initially, as a complementary tool to assist the clinical work up on a hypothesis of a particular seizure onset zone or network.

Electrical Impedance Tomography is an imaging technique with which internal conductivity changes are reconstructed from the boundary measurements (Holder 2005). These measurements are made by injecting current between a pair of electrodes and recording the voltages at all available contacts. EIT has a unique potential to advance the management of epilepsy by imaging changes occurring over milliseconds (‘fast changes’, due to ion channel opening, up to 1% impedance change) or seconds (‘slow changes’, due to cell swelling as a consequence of intense depolarization in the epileptogenic region, up to 10% impedance change) (Aristovich et al., 2014; Vongerichten et al., 2016; Hannan et al., 2018). The study presented here aimed to record slow impedance changes. Due to the larger signal generated, as well as a different EIT system design (Dowrick et al., 2015), slow impedance changes can be recorded as single shot imaging of each ictal event. The images can be generated at similar to fMRI time resolution (fMRI-like) but without limitations of fMRI, such as requiring seizures on demand, using expensive, well-isolated and large equipment, and generating a large ictal movement artifact. Currently, EIT requires the use of intracranial electrodes (Fabrizi et al., 2006) but it can be attached to contacts already used in the presurgical evaluation of epilepsy patients i.e. as combined simultaneous intracranial depth electrode and Electrical Impedance Tomography monitoring (SEEG-EIT). SEEG-EIT is a method proposed to work in cooperation with a clinical hypothesis, so that it can operate on any electrode configuration available. Although some electrode arrangements, for instance bilateral, would increase EIT sensitivity for the whole head imaging, there is currently no requirement to where the electrodes are placed for EIT to work.

The feasibility of its use clinically in epilepsy patients has been evaluated in a modeling study with real patient data, in which EIT was found to improve localization of seizure onset when compared with SEEG and inverse source modeling (Witkowska-Wrobel et al., 2018). Thus, there is a clear rationale to follow this *in-silico* study with an *in-vivo* study to confirm efficacy of SEEG-EIT in epilepsy assessment prior to moving the technique to clinical trials.

Since the porcine brain resembles the human brain in size, presence of gyri, gross anatomy, degree of myelination and development (Van Gompel et al., 2011; Sauleau et al., 2009), it provides a valuable neurological research model. Its size suggests it should be also well suited for SEEG with human electrodes mirroring the human brain in a clinical situation even better. However, the development of seizure or epilepsy models in the pig has been limited. One model that has been tested across species and found to be reliable is a chemical seizure induction with penicillin-triggered epileptogenic activity (Van Gompel et al., 2011,^2014^; Mäkiranta et al., 2005; Leaming et al., 1999, Terndrup et al., 1999,^1994^,^1995^). In this model, a single cortical injection of penicillin induces recurrent focal epileptogenic activity. The focal reduction of GABA-dependent inhibition caused by penicillin results in an increase in excitatory cortical afferents, and therefore triggers epileptiform bursts (Fisher, 1989). Given potential applicability of this model to the pig as well as both the similarity of the porcine brain to the human one and the possibility to use human SEEG electrodes in it, the swine model of epilepsy seem perfectly suited for testing efficacy of SEEG-EIT *in vivo*.

### Purpose

The purpose of this study was to determine if a real time parallel EIT system could produce accurate images of seizure onset and propagation in the porcine brain. This was evaluated in a chemical model of epilepsy (focal injection of benzylpenicillin) in the anaesthetized pig using intracranial electrodes. The questions to be answered with this work were as follows:

Does EIT have the ability to image the onset and propagation of the seizures in real time?

If so, what is the accuracy of the method?

What are the limitations of the method?

### Experimental design

**Fig. 1:**
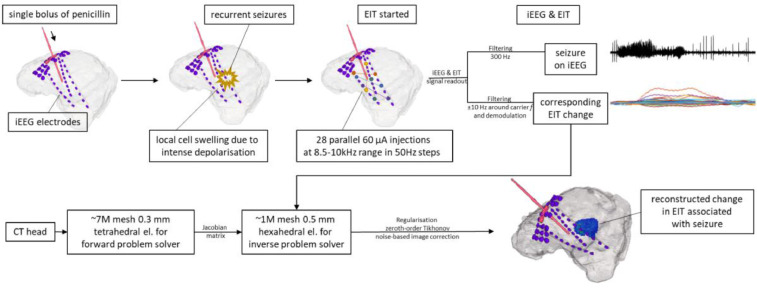
Overview of the technique used in this study. Top row: pig's brain was implanted with iEEG (intracranial EEG: SEEG and ECoG) and cannula for penicillin. A single bolus of procaine benzylpenicillin was delivered at the beginning of each experiment to create an epileptogenic lesion and consequently trigger repetitive seizures. EIT injected 60 µA current simultaneously between 28 pairs of SEEG-SEEG and SEEG-ECoG contacts during the whole experiment. Each injection pair was set at different frequency (f) between 8.5 and 10 kHz, in steps of 50 Hz. The recorded signal was a mixture of iEEG and impedance change (dZ) extracted with appropriate filters. Repetitive seizures seen on iEEG (top right corner, top trace) caused regional cell swelling due to intense depolarization, which was then picked up by a corresponding, but delayed, EIT signal change (top right corner, bottom trace) seen on all recording channels. Bottom row: after the experiment, CT head was used to create a tetrahedral mesh with accurate locations of iEEG electrodes for each individual animal to solve the forward problem and create the Jacobian (sensitivity) matrix. Then, a coarser hexahedral mesh was also made and used to solve the inverse problem. Subsequently, data were regularized, and noise corrected. The final image (bottom right corner) shows a snap of the reconstructed impedance change inside pig's brain associated with the on-going seizure. Such reconstructed EIT images can be investigated over time to demonstrate change during the seizure progression in real time.

EIT was performed in a large animal model during a specifically developed acute focal model of chemically induced seizures under general anaesthesia. Brain activity was monitored with human ECoG and SEEG. Seizures were triggered with a focal intracranial injection of procaine benzylpenicillin (BPN). The EIT was recorded with a parallel EIT system measuring slow impedance changes occurring over seconds, simultaneously to intracranial EEG (Fig. 1). The experiments were performed on five pigs. The BPN epilepsy model resulted with 205 focal, focal secondary generalized and generalized seizures. Raw impedance signal change was calculated for all seizures. Focal and focal secondary generalized seizures (37/205) were observed in three pigs and these were reconstructed into EIT images, as they could be compared objectively with the ECoG/SEEG for the onset and seizure spread. Image reconstruction was performed with subject specific FEM meshes for forward and inverse calculations using zeroth-order Tikhonov regularization, automatic cross-validation for hyperparameter selection and noise-based image post-processing (Aristovich et al., 2014). 3D EIT images had one-millimetre resolution of every second around the seizure onset. The location of the onset and spread of the impedance change during seizures was evaluated by comparing to electrophysiological findings.

## Materials and methods

### Animal preparation and surgery

All experimental procedures and investigations performed during this study were ethically approved by the UK Home Office and undertaken under its regulations, as described in the Animals (Scientific Procedures) Act 1986 and EU Directive 2010/63/EU.

Five pigs (70–75 kg, 4-month-old females) were used for simultaneous SEEG-EIT recordings. For all animals, the anaesthetic protocol included induction with ketamine and midazolam. All pigs were then intubated and mechanically ventilated; anaesthesia was maintained using sevoflurane. Respiratory rate, heart rate, invasive arterial blood pressure, SpO_2_ and rectal body temperature were monitored regularly. All animals were given pancuronium (initially 0.1 mg/kg and up to 0.08 mg/kg as needed) to maintain muscle relaxation after seizure induction.

The pig's head was secured into a stereotaxic frame (Model 1430, Kopf Instruments). Lidocaine was injected subcutaneously within the region of the craniotomy. The skin was incised longitudinally over the left side of the dorsal cranium, and subcutaneous tissues reflected. Left fronto-parietal bones were removed with an air drill to expose the cerebral cortex. The cranial window was approximately 5 cm in length and 2 cm wide, extending from 1 cm caudal to the frontal-parietal suture to the parietal-occipital suture, with a lateral boundary of around 0.5 mm from the superior edge of the orbit, forming a trapezoidal opening. Due to the significant thickness of the pig skull at this age (20–30 mm), additional grooves were drilled at the posterior edge of the opening to facilitate passage of the array connecting leads. The dura was incised to expose the somatosensory cortex. Prior to opening the dura, a bolus of mannitol (0.5  g/kg) was injected intravenously to ease epicortical array placement.

**Fig. 2:**
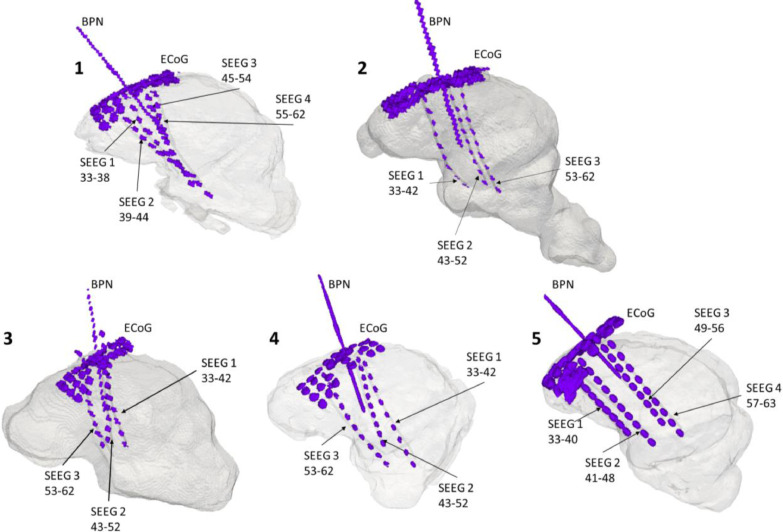
Brain and electrode FEMs for all 5 animals tested in this study. The precise location of the electrodes and cannulas were obtained from animal-specific CT performed at the end of the recordings. Labels: ECoG – cortical brain activity recorded with the epicortical array with 32 ECoG contacts; SEEG – deep brain activity recorded with depth electrodes with the respective numbers specifying how many contacts were collecting data on each probe; BPN – the site where the cannula for benzylpenicillin injection was placed with respect to the electrodes.

A subdural grid array electrode (AdTech, Severn Healthcare Technologies, UK) with 4 × 8 platinum contacts, 10 mm spacing, 4 mm diameter, 2.3 mm exposure, 2 tail exposure grid was manually placed over the left somatosensory cortex with its edges secured under the edges of the craniectomy window. Four holes in the array were pre-prepared in advance to create entry points for the depth electrodes. Depth electrodes were implanted manually, directed towards the midline, hippocampus, amygdala, and thalamus. Electrode placement was chosen to mimic a clinical situation, in which intracranial EEG signal is collected from and around the most likely epileptogenic focus or network but there was no anatomical distinction made to guide further analyses. Three to four depth electrodes were used in each animal, according to brain size and geometry. Depth electrodes were platinum, 10-contact grids of 0.86 mm diameter and 2.29 mm recording length per contact, with 5 mm spacing (AdTech, Severn Healthcare Technologies, UK). A 20 G cannula for benzylpenicillin injection was inserted through the center of the array, again directed towards the midline, keeping in line with the depth electrodes (Fig. 2).

Plain film radiographs (dorsoventral and left lateral views) were taken after electrode and cannula implantation for initial verification of appropriate positioning. At the end of each experiment, animals were euthanized with an overdose of pentobarbital and computed tomography (CT) of the head was performed.

### Induction of seizures

Seizures were induced with a single intracranial injection of 6000 IU (20 μL) of procaine benzylpenicillin (BPN, Depocillin, 300 mg/ml, Intervet). BPN was delivered via an infusion pump through a 20 G plastic cannula placed through a hole in the epicortical array over the somatosensory cortex in all experiments. The BPN cannula was inserted between depth electrodes but it was not aimed specifically at any structure to avoid introducing any bias in the reconstruction. This triggered cyclic epileptiform activity in three pigs; an additional 10 or 30 μL was required in two pigs before epileptiform activity was seen. These further doses were given within 60 min of the initial BPN dose. Otherwise, there was no additional injection given that could cause repetitive regional swelling. Cyclic epileptic activity continued spontaneously for the following three to four hours until the pigs were euthanized. ECoG and SEEG signal was recorded throughout the experiment.

### Data acquisition

Simultaneous ECoG/SEEG and EIT recordings were made from 63 channels at a sampling frequency of 50 kHz, with a 128-channel ActiCHamp EEG system (Brain Products GmbH) and a hardware antialiasing filter (cut-off frequency of 12.5 kHz). No software filters were set during data acquisition. The recording was undertaken with all 32 contacts on the ECoG array and 30–32 channels on SEEG.

EIT was set to inject 60 µA at 32 electrode pairs in parallel using a frequency division multiplexing Electrical Impedance Tomography, FDM-EIT (Dowrick et al., 2015; Avery et al., 2017,^2019^), over a 8.5–10 kHz range, separated into 50 Hz steps. Independent voltage measurements were made on all of the electrodes at the same time, allowing performing a single-shot data collection and, by so, reconstructing images of every ictal event. There were 28 independent current injections yielding 1548 measured voltages per animal. The injecting current contacts were preselected creating a protocol to intersect the region of an epileptiform lesion created with a BPN injection. The current injecting pairs were always formed between different SEEG electrodes, ECoG and SEEG, or ECoG and ECoG but never between the contacts on the same SEEG electrode. Following such current injection protocol allowed wide coverage of the region of interest but remained within the safety regulations and limits (IEC 60601-1 and BS5724). EIT recordings were started once all four grades of epileptogenic activity were repeated three times. The preliminary recording of the repetitive intracranial EEG pattern was used as an objective control for a formation of an established epileptogenic lesion.

The ECoG/SEEG was filtered at 300 Hz (low pass, 3rd order). EIT images were generated from the modulus of the complex impedance, filtered, and demodulated with a bandwidth of ±10 Hz around each carrier frequency (30^th^ order IRR filter). This step was necessary to separate single frequency signals from each other before the demodulation. After demodulation, the signal-sampling rate was reduced 50 and 500 times for EEG and EIT signals, respectively. An additional band-pass filter with the bandwidth between 0.01 and 1 Hz (1st order Butterworth) was used for the EIT data prior to image reconstruction. This was applied to improve the SNR after evaluating the initial results that the seizure time course for all seizures was between 1 and 100 s.

### 2.4. Data analysis

#### 2.4.1. Raw ECoG/SEEG and impedance signal analysis

The ECoG/SEEG and impedance change (dZ) were divided into blocks containing a single seizure with 30 s prior to and 60 s after the ictal event. Using a wide block of time around seizure provided a control for further EIT signal analysis. The ECoG/SEEG onset and the number of contacts involved in the seizure were established by visual assessment of all traces, as performed in clinical EEG reporting. This was further confirmed with automatic RMS thresholding (detailed explanation in ‘ECoG/SEEG and EIT correlation’, Section 2.4.3.).

The dZ is defined by applying a bandwidth filter around each carrier frequency and demodulating the filtered signal. The dZ baseline was calculated as the mean amplitude of the signal over the first 20 s prior to each seizure. The dZ significant change was expressed by comparing the dZ value with the dZ baseline at each time point using a paired *t*-test (*p* =0.01). Excessively noisy channels were identified by calculating the standard deviation of the baseline. Channels with standard deviation of the baseline of above 5 μV (dZ) or 50 µV (ECoG/SEEG) were rejected from further analysis. These accounted for less than 10% of the total number of channels.

The impedance change was described in volts, which referred to the voltage change recorded with constant current considering that they were proportional, and as a percentage change from the baseline. The maximum dZ change was characterized by finding the maximum voltage change across all significant channels for a seizure. The dZ onset was described by the time point at which the channel with maximal dZ change reached double the baseline noise for this channel and this was followed by a dZ increase lasting longer than 5 s. Raw dZ changes were analyzed for all 205 seizures recorded in five animals.

A focal seizure was classified as a local ictal discharge, originating within limited networks, for which a clear SEEG onset could be distinguished from the background activity. The SEEG onset was identified as synchronized polyspikes or paroxysmal sharp waves at a frequency of more than 3.5 Hz, appearing at 2–4 contacts close to the BPN cannula and lasting at least 15 s. As depth electrodes surrounded the cannula, it was expected that the focal onset could be detected locally at all SEEG electrodes adjacent to the penicillin injection point. The onset was then followed by the spread over the majority of ECoG and SEEG contacts (called ‘focal secondary generalized’ below) or ictal activity remained within the local 8 to 12 contacts (called ‘focal seizure’).

#### 2.4.2. Image reconstruction

EIT images were produced with realistic animal-specific head meshes segmented from individual CT scans undertaken at the end of each experiment. The segmentation included two layers, brain tissue and electrodes (Jehl et al., 2016). Tissue conductivity was assumed to be isotropic and was modeled as a homogenous section of 0.22 S/m conductivity for the whole brain (Horesh, 2006). The realistic geometry of intracranial electrodes and BPN cannulae were modeled from the CT scans. The resulting meshes comprised 6.8 to 8.2 million, approximately 0.3 mm tetrahedral elements. The forward solution was calculated using the PEITS forward solver (Jehl et al., 2014). The Jacobian matrix was used for inversion, consistent with a linear one-step time difference reconstruction. The inverse problem was solved using coarser hexahedral meshes of 0.5 mm element-size and 0.5 to 1 million hexahedral elements. The reconstructed conductivity changes for each hexahedral voxel were regularized with zeroth-order Tikhonov, corrected with a noise-based variance weighting and expressed with *t*-score (*s*, sigma) for imaging (Aristovich et al., 2014). Detailed image analysis was performed for 37 seizures that presented a distinct onset in SEEG (‘focal’ and ‘focal, secondarily generalized’). The forward and inverse problems were calculated as outlined in detail in our recent study (Witkowska-Wrobel et al., 2018). Calculations were performed on a research workstation (Dell Precision, Dell Inc., UK, 256GB RAM, 16 Intel Xeon CPU cores). It took up to 150 min to compute the forward solution and about 10 to 20 min for the inverse solution per seizure. Typically, a seizure contained approximately 300 time points.

The quality of the reconstructed images was assessed with a seizure onset localization error, expressed in millimetres. This metric was defined as the distance between the center of mass of the reconstructed change at the maximum dZ increase from the coordinates of the three real onset points. These points were: the tip of BPN cannula, the mean position of all SEEG electrodes detecting the onset and the closest SEEG contact on which the onset could be detected. EIT onset reconstruction error was expressed as the mean ± 95% confidence limits, as determined by the two-tailed t-student test.

#### 2.4.3. ECoG/SEEG and EIT correlation

*Seizure onset*: ECoG/SEEG onset, i.e. the number and position of the onset contacts, was assessed visually. The number of ECoG/SEEG contacts was correlated with the maximum amplitude of impedance change for a seizure (the so-called ‘peak dZ’).

*Seizure spread*: Seizure spread over the ECoG/SEEG (‘ECoG/SEEG spread’), i.e. the number and position of the contacts detecting the seizure at its most generalized time, was assessed visually, as for the onset. The ‘EIT spread’ was expressed as the number of channels to which the EIT reconstructed change spread and covered, at 65% threshold of the maximum reconstructed value. The 65% threshold was computed based on finding an average threshold given that there were no active voxels before and after the region of seizure, as identified by the iEEG analysis.

The ‘ECoG/SEEG spread’ was correlated with the ‘EIT spread’.

In addition, the ECoG/SEEG-EIT correlation was calculated for the magnitude of the signal (‘peak SEEG’) with the maximal amplitude of dZ change (‘peak dZ’). The ECoG/SEEG signal magnitude was calculated from the electrographic spiking activity, by using the RMS (root mean square) of the signal on the contact on which the seizure was observed to start. The signal spectrum was obtained from the spectrogram of that channel, using a short-time Fourier transformation of the input signal. The peaks of spiking activity were defined by using a bandwidth of 20 to 40 Hz of the rectified signal, as this is where the majority of power was. This provided an index of spiking power, and therefore a clear representation of when the seizure occurred and how frequent the spikes were.

All correlations were performed with the Pearson correlation coefficient.

All data are presented as mean ± SD.

*Qualitative analysis*: Furthermore, the resulting images of the seizure propagation were compared qualitatively with the ECoG/SEEG findings for all seizures reconstructed with EIT. This was performed to determine whether the spread of the seizure seen in electrophysiological measurements matched the observed changes in reconstructed EIT images in real time and to exclude or define potential artifacts or mismatches in EIT images. This comparison was based solely on the authors’ knowledge of EEG findings and seizure onset and spread in EEG.

### 2.5. Data and code availability

The data and code used for analysis and image reconstruction are available from the corresponding author on request.

## 3 Results

### 3.1. Seizure activity in a porcine model

Initial interictal activity was observed 3 to 22 min (12 ± 7 min, mean ± SD) after BPN bolus injection in all five pigs, with the first seizure occurring after 20 to 60 min (31 ± 17 min) in three pigs. In two animals requiring an additional BPN dose, the first seizure occurred 11 and 7 min after the second (and final) BPN dose. A dose of 6000 IU of procaine benzylpenicillin was the lowest effective for generating spontaneous recurrent epileptiform activity. In total, 205 seizures were recorded with EIT in five animals with 20 to 82 seizures per animal. Three seizure patterns could be differentiated in ECoG/SEEG: focal, with a clear ECoG/SEEG onset (‘focal seizures’, *n* =23 in three pigs), focal that secondarily spread over the adjacent contacts throughout the following 1–4 s (‘focal, secondary generalized seizures’, *n*=14 in two pigs). The rest of the seizures were generalized (*n*=154 in all five pigs) or with the onset difficult to differentiate in ECoG/SEEG (*n*=14).

In three pigs, a substantial increase in heart rate (up to 220 bpm) and blood pressure (up to 200/130 mmHg) was observed during seizure activity. The observed rise in blood pressure was less prominent and progressed more slowly than the heart rate increase. This was not related to administering an additional BPN dose.

**Fig. 3:**
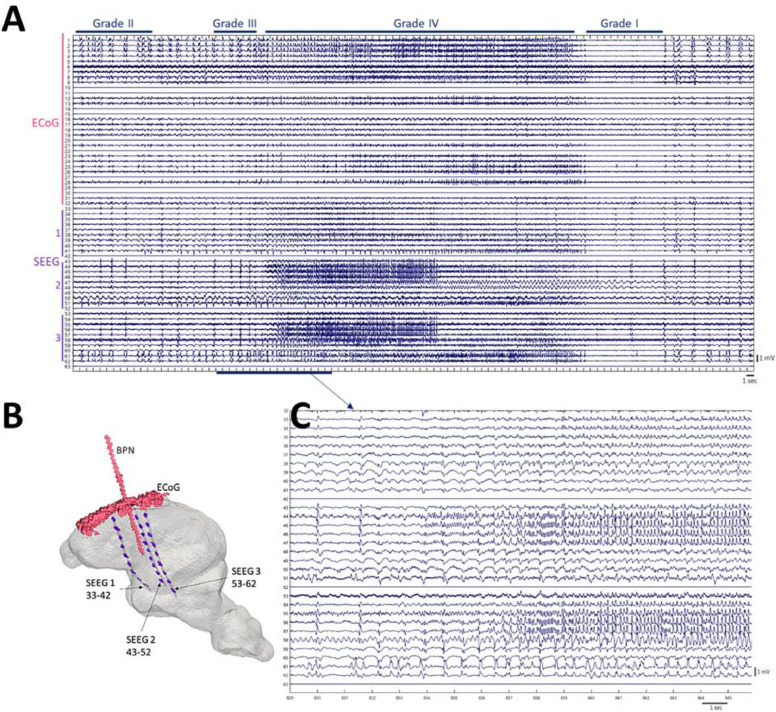
Seizure activity in porcine model. Panel A: representative example of a full cycle of epileptiform activity recorded with a 32-electrode array ECoG and 3 SEEG 10-contact depth electrodes. The epileptiform grades are listed above the EEG panel. Evolution of four epileptiform grades is presented over 100 sec throughout all electrodes with an expanded view of SEEG onset over 15 sec (Panel C). Panel B: head-model illustrates the electrodes and BPN cannula coordinates. The numbering of SEEG contacts follows the rule that smaller numbers are located more ventrally/deeper within the brain, with progressively higher numbers moving towards the surface of the brain (i.e. for SEEG 1, contact 33 is the deepest and contact 42 is in the cortex).

Epileptiform cycles repeated every 3 to 10 min (representative example of a whole cycle in ECoG and SEEG is shown in Fig. 3 A). Epileptiform activity occurred in grades and spontaneously cycled from one grade to another, as previously reported (Van Gompel et al., 2014,^2011^;Mäkiranta et al., 2005; Stypulkowski et al., 2011; Opdam et al., 2002). The cyclic epileptic activity could be distinguished in all animals on ECoG/SEEG, switching between stable background activity (grade I), interictal spikes (grade II and III) and seizures (grade IV). Grade I, background activity lasted for 2 to 86 s (13 ± 9 s) from the end of the previous seizure. Following this, Grade II and III activity occurred with increasing interictal spike (IIS) frequency. Grade IV activity (seizures, example in Fig. 3 C) occurred spontaneously and lasted 8 to 70 s (25.8 ± 13 s, *n*=205 in 5 pigs).

### 3.2. Raw impedance responses during all seizures

**Fig. 4:**
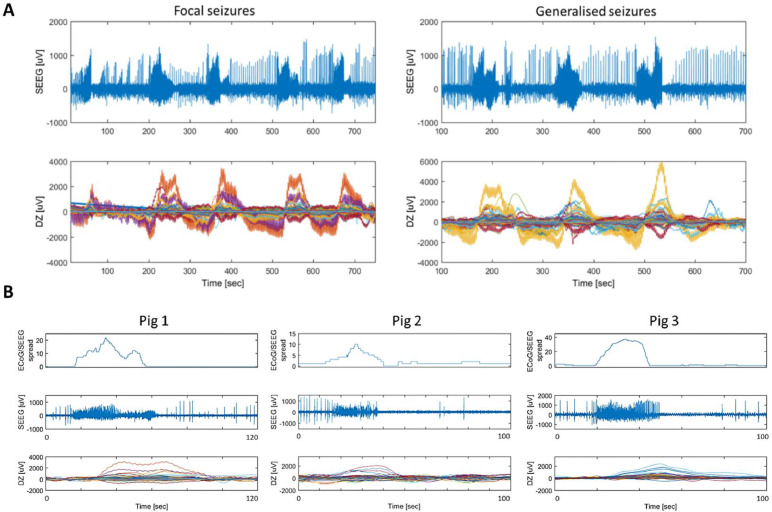
Representative examples of raw impedance increase observed during seizures. Panel A: upper graphs show one SEEG channel with interictal and ictal discharges for focal (left) and generalized (right) seizures, while lower graphs show the respective impedance changes on 1450 recording channels during these events over time (each color represents a different channel). A, left, focal seizures: the first focal seizure yielded up to 2 mV dZ change, followed by secondarily generalized seizures, for which the amplitude of the dZ change was higher. A, right, generalized: all three seizures were generalized, causing a larger dZ change, between 4 and 6 mV. Panel B: Top graphs indicate the number of ECoG/SEEG contacts recruited during seizure progression, the middle graphs show activity in the first SEEG channel to detect seizure activity, and the bottom graphs show all the impedance changes on 1450 individual channels during the seizure. There is a significant delay between the onset in SEEG and dZ.

Each seizure resulted in an impedance increase (Fig. 4 A) with a peak of 1.7 ± 0.2 mV (6 ± 2%) in focal (*n*=23 in three pigs), 2.3 ± 0.4 mV (8 ± 2%) in focal secondary generalized seizures (*n*=14 in two pigs) and 3.4 ± 1.5 mV (9.5 ± 3%) in generalized seizures (*n*=154 in five pigs).

The onset of the impedance increase was delayed from the seizure onset in SEEG by 11 ± 3 s for focal seizures (Fig. 4 B), by 12 ± 3 s for focal secondary generalized and by 15 ± 2 s for generalized seizures. The dZ peak was delayed by 29 ± 12 s from the seizure onset in SEEG on average. There was no impedance signal change detected between the seizures.

### 3.3. EIT images

**Fig. 5:**
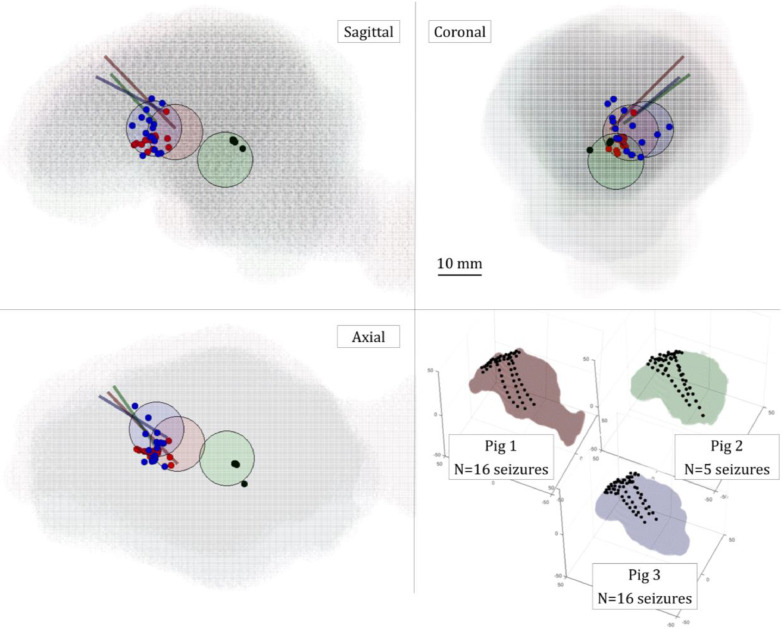
EIT onset reconstruction with respect to the ECoG/SEEG electrodes overlaid for three pigs. In all four panels, the colors are consistent for a given animal, i.e. pig 1 – red, 2 – green and 3 – blue. In overlaid panels, the brain is shown in three planes: sagittal, coronal and axial, with the whole head representation in oblique plane at the bottom right. In overlaid panels, the EIT onset reconstruction is expressed by dots of respective color for 16, 5 and 16 focal seizures in pig 1, 2 and 3 respectively, in relation to the area of SEEG electrodes detecting the onset (colored circles) and the colorful lines represent cannulae for BPN injection. All ECoG/SEEG electrodes are represented as black dots in the right bottom panel. (For interpretation of the references to color in this figure legend, the reader is referred to the web version of this article.).

In three pigs that exhibited seizures with identifiable onset (*n*=37), EIT located the initial seizure impedance change to within maximum of 10 mm of its apparent onset from the three independent methods of assessment: 9 ± 1.5 mm from the tip of the cannula, 9.7 ± 1.4 mm from the mean position of all SEEG electrodes detecting the onset, and 7.5 ± 1.1 mm from the closest SEEG electrode sensitive to the onset (Fig. 5 and Table 1, *n*=37). There were no differences in onset localiZation for just focal vs. focal-generalized seizures (Fig. 5).

**Table 1:** Accuracy of EIT onset reconstruction. ‘EIT onset reconstruction’ columns show the n number of all focal seizures reconstructed with EIT for each pig and the distance accuracy of the EIT onset detection with respect to the coordinates of three points, expressed in mm. These points were: ‘cannula tip’: the position of the tip of cannula used for benzylpenicillin injection, ‘mean SEEG’: the mean position of all SEEG active electrodes at onset, ‘closest SEEG’: the closest SEEG electrode detecting seizure onset. The ‘Real location’ columns present the distance between the tip of the BPN cannula and the mean position of all SEEG active electrodes at the onset (‘cannula to mean SEEG’) and the closest SEEG electrode detecting the onset (‘cannula to closest SEEG’). The EIT onset reconstruction error is expressed as mean ± 95% confidence limits, as determined by two-tailed *t*-student test.

EIT onset reconstruction [mm]				Real location [mm]	
Animal	Cannula tip	Mean SEEG	Closest SEEG	Cannula to mean SEEG	Cannula to closest SEEG
Pig 1 (*n* =16)	6.9 ± 1.0	6.5 ± 0.9	4.8 ± 1.3	4.7	3.9
Pig 2 (*n* =5)	19.1 ± 2.0	5.5 ± 1.4	7.1 ± 2.7	19.5	13.9
Pig 3 (*n* =16)	7.8 ± 1.0	14.1 ± 1.0	10.1 ± 0.8	11.2	16.8
Overall (*n* =37)	9 ± 1.5	9.7 ± 1.4	7.5 ± 1.1		

### 3.4. ECoG/SEEG and EIT correlation

**Fig. 6:**
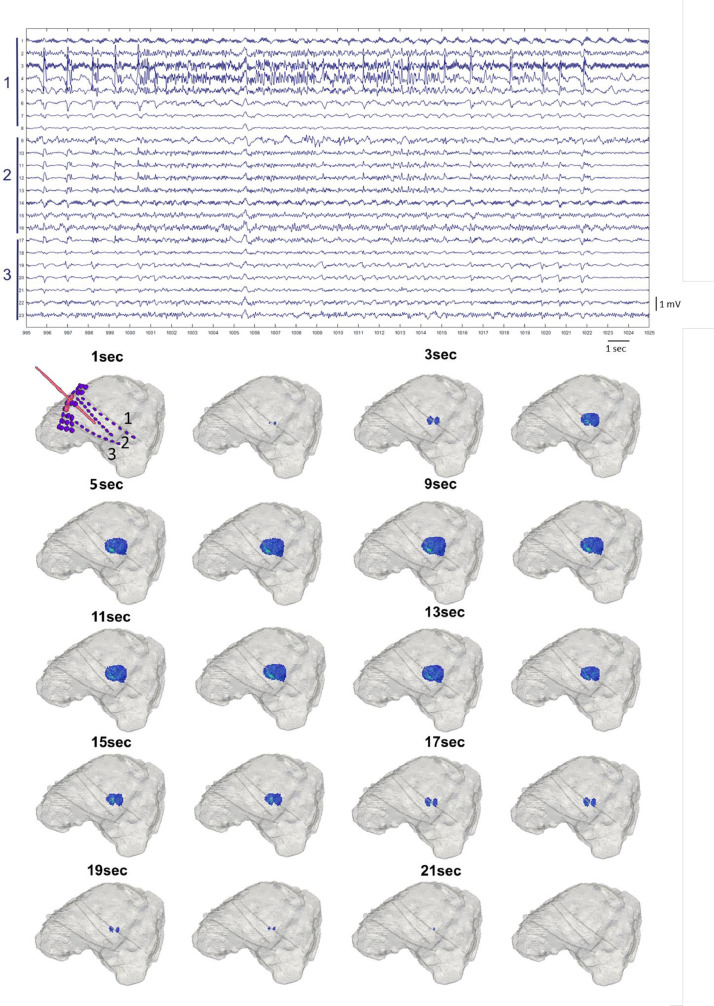
Example of progression of a focal seizure over SEEG (top) and EIT images (bottom). Top: representative example of a short focal epileptiform activity recorded with 3 SEEG depth electrodes. Bottom: EIT reconstructed onset and spread of the seizure. The dZ change started 12 s after the SEEG onset. The regional spread among the adjacent contacts seen on EIT images correlated with SEEG findings. The reconstructed EIT images are shown at every second resolution with one image between 5 and 9 s representing change seen at 6 to 8 s. This is due to reconstructed change remaining unchanged.

EIT images correlated qualitatively to within 1 cm to changes observed in ECoG/SEEG recordings as seizure activity progressed in 37 reconstructed seizures with a lag of 11 ± 3 s (representative SEEG-EIT comparison Fig. 6).

**Fig. 7:**
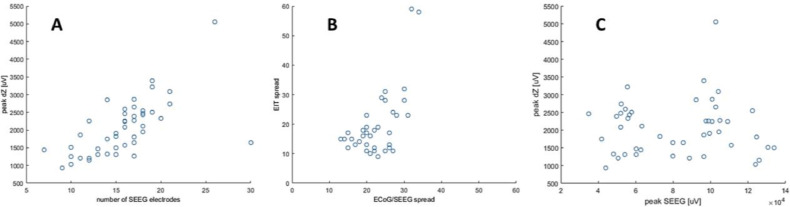
Correlation between ECoG/SEEG and EIT signal. (A) Correlation between the number of contacts involved in seizure onset on ECoG/SEEG channels (during first four seconds of the onset, x-axis) with the amplitude of the impedance change (peak dZ, y-axis). (B) Correlation between the number of contacts involved during the maximal spread of the focal seizure on ECoG/SEEG (x-axis) with the number of ECoG/SEEG contacts to which the seizure spread on EIT reconstruction (y-axis). (C) No correlation between the spiking magnitude (peak SEEG, x-axis) with the amplitude of the impedance change (peak dZ, y-axis).

The amplitude of the impedance change positively correlated with the increasing number of channels on which the seizure started (*r* =0.6, *p* <<0.0001, Fig. 7 A) and to which it spread (*r*=0.6, *p* <<0.001, Fig. 7 B) but not with the intensity of spiking activity seen on SEEG (*r=*0.03, *p*<0.9, Fig. 7 C, *n*=37 seizures in three pigs).

## 4 Discussion

### 4.1. Summary of results

This study has shown that a single intracranial injection of BPN lead to a recurrent cyclic pattern of focal and generalized seizures in the porcine brain (Fig. 3). Each seizure caused an impedance increase detectable with the parallel EIT system. These impedance changes occurred with a delay compared to the electrophysiological onset of the seizure as detected with intracranial electrodes (Fig. 4). The magnitude of the impedance change correlated with the spread of the seizure on ECoG/SEEG (Figs. 5 and 7).

When the detected impedance change was reconstructed, the produced images showed a correlation between the SEEG and the position of the tip of the cannula for seizure onset and with the ECoG/SEEG for seizure spread (Table 1, Figs. 5 and 6). This allowed imaging the seizure and tracking it in near real time.

### 4.2. Technical considerations

The overall computational time and memory usage of the reconstruction involved up to 5 h of CPU time per seizure. Still, once the mesh had been created and the sensitivity matrix calculated, this could be reused for all further seizures in the same animal, and so a reconstruction of a single seizure took between 15 and 40 min (depending on the length of the seizure) for 64 injection pairs. In this study, we used automatic hyperparameter evaluation for each image reconstruction. However, we have noted that for all cases the optimal hyperparameter showed minimal variation, which suggests that it can be pre-evaluated and fixed. In this case the image reconstruction can be performed in real time as it resembles a simple matrix multiplication. This suggests that this method could be feasible for use in clinical settings of telemetry monitoring of epilepsy patients if all necessary initial computations are performed at the beginning of the study.

### 4.3. Does EIT have the ability to image the onset and propagation of the seizures in real time?

EIT results were compared with electroencephalographic representation of seizures, assuming that seizures are the gold standard for presurgical epilepsy assessment rather than an epiphenomenon. The EIT signal was delayed from the SEEG onset but it followed the pattern of the seizure seen with electroencephalography. Due to the nature of slow EIT signal, arising over seconds as a result of changes in the extracellular space, such delay was expected (Holder 2005). The raw impedance changes were very consistent, providing the impedance increase without averaging for every seizure recorded. The magnitude of the dZ correlated with the spread of the seizure on ECoG/SEEG, resulting in the largest impedance changes measured for generalized seizures. Such changes allow reconstruction of each seizure in a real time mode so that the onset and spread can be followed with a resolution of one second. The ictal images are comparable with the discharges seen over the ECoG/SEEG electrodes for the onset, regardless of the position of the BPN cannula. This was especially striking for the second animal with focal seizures in which the recorded SEEG changes and the apparent onset of the EIT reconstruction both pointed to the very same area around 2 cm away from the tip of the cannula. For each animal with an electrophysiologically clear onset zone, the EIT reconstruction consistently identified very similar brain regions as the potential foci.

The overall accuracy of reconstruction for seizure onset in all animals was below 1 cm from the cannula or the area of activity on SEEG. These results clearly show that EIT can provide accurate real time imaging of the onset of focal seizures using intracranial electrodes. The work is extremely promising for further clinical applications, as the data analyzed here were collected with a setup currently in use in the clinic that provides both SEEG and EIT recordings concurrently.

### 4.4. What are the limitations of using EIT for imaging seizures *in vivo*?

In its current state, EIT as a method has some limitations. The main drawback is that EIT is currently limited to invasive recordings only. Other imaging methods used in the epilepsy field, such as SPECT, PET or EEG-fMRI do not require intracranial electrodes and can be used in presurgical evaluation prior to invasive SEEG implantation. EIT, however, requires intracranial electrodes to obtain sufficient signal-to-noise ratio to allow detection of changes while injecting safe levels of current. Hence, it cannot be used during routine EEG recordings. However, EIT still holds a significant advantage in its ability to record in parallel with intracranial monitoring, while using only the SEEG electrodes already implanted for clinical reasons and little additional and fully battery-powered equipment. EIT can be used continuously with video telemetry during both interictal and ictal events, not requiring seizures on demand and recording independently from their time of occurrence. This could be especially beneficial for patients with nocturnal events or episodes following falling asleep/just before waking up or for patients, who require prolonged monitoring because their seizure episodes are simply rare. All such events are difficult, if not impossible, to record with ictal-SPECT, PET, and fMRI. Also, in case of EIT-SEEG monitoring, there is no need for special patient preparation, for trained personnel to be available at a particular time slot (for example to inject a tracer at the beginning of a seizure as required in ictal-SPECT), or for any expensive equipment with dedicated room for it.

In addition, this study is limited by using a combination of two intracranial EEG recording methods that are rarely used together in patients. However, both types of intracranial electrodes, ECoG and SEEG, had to be combined to achieve an adequate number of recording contacts, similar to that encountered in the clinical setting in a much larger human brain with more depth electrodes.

In this study, EIT has been shown to be capable of providing 3D images of the focal seizure signal spread over the brain that correlates with the spread over the ECoG/SEEG. However, here, the seizure onset was located exactly between the SEEG electrodes; therefore, the majority of current injections, and consequently the highest sensitivity of EIT, were all focused around the onset area. This is unlikely be the case in clinical patients, for whom the electrodes are more widespread, covering distant areas between cerebral lobes or even between hemispheres. Despite this, the results for imaging of the spread of focal seizures with EIT in this setup suggest that EIT could potentially be used to define the margins of the epileptogenic zone. This would be extremely beneficial in cases with large lesions, for whom it is impossible to surgically resect the whole dysplastic region. Another potential application for EIT could be lateralizing the onset, for instance in patients who have bilateral hippocampal lesions/sclerosis or identifying primary and secondary epileptogenic zones in patients with multifocal abnormalities. The latter could be cases of tuberous sclerosis and multiple tubers, or cortical developmental dysplasia, such as nodular heterotopias. EIT could potentially also offer imaging support in some cases of focal cortical dysplasia for which MRI and EEG findings are sometimes very subtle. Finally, EIT could be used as a support for patients and with previous resections with misleading scalp EEG. However, further work is now required to assess whether EIT can be used to distinguish such epileptogenic zones within the dysplastic region of the cortex and if so, to determine its accuracy.

Only focal seizures were reconstructed in this study, based on the assumption that they would best represent the typical clinical scenario. In general, only patients with focal refractory epilepsy are further investigated as potential candidates for resective surgery. Focal seizures can be evaluated with EEG, which is currently the gold standard method for seizure assessment. EIT could then be compared with the EEG findings to optimize precision of seizure focus detection. If seizures are generalizing rapidly, it is difficult and often impossible to define the epileptogenic zone and, consequently, to decide where to implant intracranial electrodes and where to subsequently operate. Hence, we assumed that it would be significantly less likely that EIT would be performed in the hospital setting in patients with generalized seizures. While we were able to record many generalized seizures in our animal study, including the two pigs that demonstrated only generalized seizures throughout the whole experiments, EIT imaging accuracy of the onset and spread of generalized seizures was not analyzed further, due to its lack of direct clinical relevance and absence of potential application in the clinic as well as the difficulty in comparing any potential results of such analysis with the EEG data.

Finally, slow impedance changes are reported to occur due to cell swelling during local intense depolarization and concurrent shrinkage of the extracellular space due to cellular uptake of water and excess potassium ions (Holder 2005). Such changes arise over seconds and should follow neuronal activity, whereas the EEG has a resolution of milliseconds and detects a summation of postsynaptic activity, both inhibitory and excitatory (Smith, 2005). However, the results seem to indicate that in the current setup EIT is capable of repeatable and accurate localization of seizure onset that is consistent with SEEG findings and could aid clinical decision-making.

### 4.5. Conclusions and future work

This is the first time that EIT has been used in a large, folded brain with human intracranial electrodes during ictal events. Seizures induced with procaine benzylpenicillin produced cyclic epileptic activity that could be easily followed and distinguished in ECoG/SEEG. The parallel EIT system provided a reliable method for accurate localization of the onset of the focal seizures when compared with the ECoG/SEEG outcome. The method offers exciting potential to follow a spread of a seizure in the near real time and may improve the localization of seizure foci in clinical patients. The most important advantage of EIT is that it can be already used in parallel with intracranial electroencephalography while recording all ictal and interictal activity. EIT produced functional images showing ictal spread with a temporal resolution of seconds, with a spatial accuracy of less than one centimeter from the onset area defined either by the tip of the cannula or by the active SEEG electrodes. However, at current design, it is impossible to specifically determine EIT resolution for the whole brain imaging. This is due to several factors that contribute to EIT sensitivity, such as electrode placement, number of contacts used for current injections and potentially the complexity of patient's epilepsy.

The correlation in seizure spread between ECoG/SEEG and EIT was only performed at a single time point (when the seizure resulted in the maximum generalized activity on ECoG/SEEG). This work should be extended to compare the volume of the EIT change with the ECoG/SEEG findings over time. These findings could be also extended by parallel studies with fMRI. Future work should additionally focus on establishing EIT accuracy if the seizure onset is located further from the SEEG electrodes, as well as potentially analyzing generalized seizures in greater detail. Finally, there is a need to better understand the coupling between interictal spike activity in EEG and the EIT changes and the location of the primary focus or foci. A further assessment of the impedance changes over the interictal discharges (grade II and III in described model) would be also desirable.

Overall, EIT as a method for seizure imaging requires further studies but appears to hold a potential to improve the localization of the seizure onset zone and, in long-term, to enhance understanding of epileptic circuits and assist seizure therapies. Also, the findings of the presented study indicate that the method, at its current state, could be directly taken into preliminary human trials for recording data from patients with epilepsy and intracranial electrodes.

## Data and code availability

The data and code used for analysis and image reconstruction are available from the corresponding author on request.

## Author contribution statement

**Anna Witkowska-Wrobel**: Conceptualisation, Data curation, Formal analysis, Investigation, Methodology, Resources, Visualisation, Writing (original draft), Writing (review and editing); **Kirill Aristovich**: Conceptualisation, Formal analysis, Investigation, Methodology, Resources, Software, Writing (review and editing); **Abbe Crawford**: Investigation, Resources, Writing (review and editing); **Justin D. Perkins**: Resources, Funding acquisition, Supervision, Writing (review and editing); and **David Holder**: Conceptualisation, Funding acquisition, Supervision, Writing (review and editing).

## Declaration Competing of Interest

None.
